# Endoplasmic reticulum protein retention and disturbed proteostasis is a common pathology for a subset of autism: evidence from mutations in GABA_A_ receptors and GABA transporter 1

**DOI:** 10.3389/fnmol.2025.1671331

**Published:** 2026-01-16

**Authors:** Jing-Qiong Kang, Aiden Delahanty

**Affiliations:** 1 Departments of Neurology and Pharmacology, Vanderbilt University Medical Center, Vanderbilt University, Nashville, TN, United States; 2 Vanderbilt Kennedy Center of Human Development, Vanderbilt Brain Institute, Vanderbilt University, Nashville, TN, United States

**Keywords:** endoplasmic reticulum, GABA_A_ receptors, GABA transporter 1, proteostasis, autism, epilepsy

## Abstract

Autism is a common childhood disorder, often comorbid with epilepsy. Both autism and epilepsy are highly heterogeneous in terms of disease etiology and frequently co-occur with other neuropsychiatric phenotypes. Advances in genetic sequencing technologies have significantly improved our understanding of the biological pathways involved in these disorders, particularly in genetic epilepsy (GE). One critical pathway involves gamma-aminobutyric acid (GABA), a key neurotrophic signal during early brain development. GABA plays a central role in maintaining neural excitatory-inhibitory balance, and its dysfunction has been implicated in both autism and epilepsy. GABA acts through its receptors and transporters to regulate neuronal signaling, and disruptions in this system can lead to neural circuit abnormalities. Recent studies have identified that mutations in GABA_A_ receptors and the GABA transporter 1(GAT-1) encoding *SLC6A1* result in defective protein folding and retention in the endoplasmic reticulum (ER), leading to impaired proteostasis. This common cellular defect has been observed in a subset of patients with autism and epilepsy, suggesting a shared pathogenic mechanism. We propose that ER retention of mutated proteins and impaired trafficking contribute to disease phenotypes associated with monogenic *de novo* mutations. Consequently, therapeutic strategies aimed at enhancing protein folding and trafficking, such as the use of chemical or pharmacological chaperones like 4-phenylbutyrate, may provide cross-cutting benefits for both disorders. Our hypothesis highlights the potential for a unified therapeutic approach targeting cellular protein homeostasis in genetically defined subsets of autism and epilepsy.

## Highlights

- Impaired GABAergic signaling is a converging pathway for autistic spectrum disorders.
- Mutations in GABA_A_ receptor subunit genes are associated with autistic spectrum disorder.
- Mutations in GABA transporter 1 are associated with autistic spectrum disorder.
- Endoplasmic reticulum protein retention and disturbed proteostasis are common for GABA_A_ receptors and GABA transporter 1 mutations.
- Therapeutic opportunities based on the shared endoplasmic reticulum related disease mechanisms.

## Introduction

It has been long observed that children with autism are more likely to develop seizures while children with epilepsy are more likely to display autistic spectrum traits (Lotter, 1974; Deykin and MacMahon, 1979). Genetic mutations are known to be a common underlying cause of both epilepsy and autism (Mermer et al., 2021; Wang et al., 2020; Koesterich et al., 2023; Sanders et al., 2018; Spratt et al., 2019; O’Reilly et al., 2021; Fu et al., 2022) as well as other neurodevelopmental disorders (Mermer et al., 2021). Autism and epilepsy as disease entities may involve different neuronal circuitry that give rise to distinct phenotypes at clinical level. However, based on our extensive studies on GABA_A_ receptor subunits and GABA transporter 1 (GAT-1)-encoding solute carrier (SLC) family 6 member 1 (*SLC6A1*) mutations, autism and epilepsies may have overlapping patho-mechanisms at cellular or molecular levels, which can be leveraged for novel unified development of therapeutic strategy.

**Figure 1:**
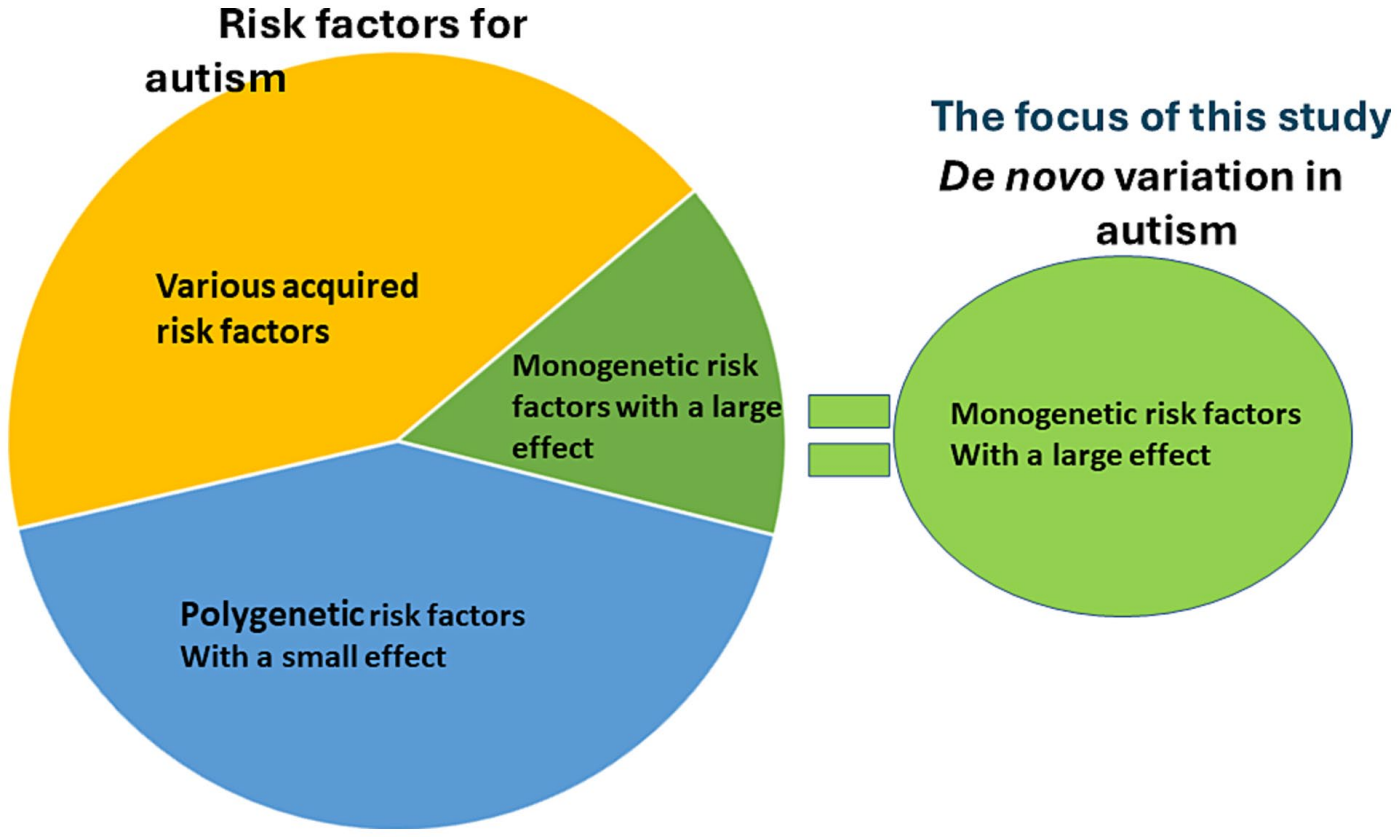
Acquired or genetic etiologies for autism. There are a wide range of acquired or environmental risk factors for autism and genetic risk factors. For genetic risk factors, there are two categories: polygenetic risk factor with a small effect and monogenic risk factor with a large effect. Based on our extensive studies on GABA_A_ receptor subunit and GABA transporter 1(GAT-1)-encoding *SLC6A1* mutations, we propose that the subset of autism associated with monogenic risk factor exist common endoplasmic reticulum (ER)-related pathology such as protein ER retention and ER stress as in genetic epilepsy.

Autism is a prominent disorder occurring in childhood that interferes with the normal course of social, communicative, and cognitive development, affecting 1 out of 150 children at a male to female ratio of 4:1 (Abrahams and Geschwind, 2008; O'Roak and State, 2008). In addition to the core deficits, autism has co-morbidity with several neuropsychiatric disorders and social difficulties (Lotter, 1974) including hyperactivity, epilepsy, cerebral palsy and cognitive difficulty, suggesting pervasive brain abnormality. Epilepsy is also a common childhood disorder with ~1–3% prevalence worldwide depending on different regions. Genetic factors also play a significant role in autism, with approximately 50–60% of cases attributable to heritable influences, primarily driven by common genetic variation (Gaugler et al., 2014). An estimated 10–20% of individuals with autism have the condition due to rare, *de novo* monogenic mutations (Sanders et al., 2015; Iossifov et al., 2014), which is a much smaller fraction of the autism patient population comparing to those common variations. The *de novo* monogenic mutations have a large effect on damaging the coded protein function. Both autism and epilepsy are highly heterogenous with regards to disease etiology, and both can be caused by acquired and genetic factors. It has been proposed that autism is associated with various environmental factors such as neonatal hypoxia, gestational diabetes mellitus, paternal age >50, preterm birth, and exposure to valproate (Lord et al., 2020). In addition to environmental factors, genetic studies in autism have identified many genetic risk factors. These include rare monogenic variation with a large effect and common genetic variation with a small effect (Sanders et al., 2018; Fu et al., 2022) (Figure 1).

This study focuses on a subset of autism that has rare *de novo* monogenetic mutations with a large effect. The subset is estimated to be 10–20% of people with autism (Lord et al., 2020) and is associated with monogenic mutations, such as mutations in *GABRB3* (Delahanty et al., 2011)*, SLC6A1* (Wang et al., 2020)*, SCN2A* (Sanders et al., 2018) and *CH2D* as well as mutations associated with Fragile X syndrome, Rett syndrome, Angelman syndrome and Tuberous sclerosis (Fu et al., 2022; Devlin and Scherer, 2012). Among this 10–20% subset of autism, some patients may share some common pathophysiology in the endoplasmic reticulum (ER). Although these genes seem unrelated, the mutations in these genes at molecular and cellular levels affect the same endogenous protein processing pathways in the ER. Treatment interventions designed against these common pathways could be beneficial for various seemingly unrelated diseases including subsets of epilepsy and autism.

These insights into common molecular pathophysiology between autism and epilepsy are mainly gained from studying gene mutations that affect GABAergic signaling pathways. We have studied multiple variants in more than 10 subunits of GABA_A_ receptors (Kang and Macdonald, 2004; Kang and Macdonald, 2016; Kang et al., 2009a,b; Kang et al., 2015) and a large cohort of mutations in GAT-1-encoding *SLC6A1* (Mermer et al., 2021; Mermer et al., 2022; Poliquin et al., 2021). Interestingly, up to date, there is no clear association between mutations in other GABA transporters, such as GAT-2 and GAT-3, with either autism or epilepsy. There is only one study that reports the polymorphism in GAT-3-encoding *SLC6A11 to be* associated with febrile seizures (Schijns et al., 2020). In the past two decades or so, our work has focused on elucidating the molecular pathophysiology of GABA_A_ receptors and GAT-1-encoding *SLC6A1* mutations. Based on our extensive studies on the molecular pathophysiology of genetic epilepsy (GE), we identified common mechanisms underlying autism and epilepsy at molecular levels, particularly with mutant protein posttranslational processing inside the ER (Mermer et al., 2021; Wang et al., 2020; Mermer et al., 2022). We now propose that there is a shared mechanism of ER protein retention in GE and at least a subset of autism with *de novo* monogenic mutations. This thus suggests that some mechanism-based treatment developed for GE could be repurposed for this subset of autism via targeting the overlapping disease mechanisms.

### Abnormality in genes is involved in early brain development and the risk factor for autism and epilepsy

Many genes enriched in embryonic and early brain have been associated with autism. For example, *GABRB3* is highly expressed in embryonic and neonatal brain. It has been reported that the head circumference is increased in autistic toddlers, suggesting abnormal cell proliferation in at least some autism (Courchesne et al., 2003). The genetic risk factors for autism range from rare *de novo* monogenic mutations in genes encoding synaptic proteins (such as contactin-associated protein-like 2, CNTNAP2; SH3 and multiple ankyrin repeat domains 3, SHANK3; and neuroligin 3, NLGN3), to copy number variations with either gain or loss of DNA segments (for example, 16p11.2 and 15q11-q13). Additionally, gross chromosomal rearrangements contribute to about 7% of autism cases (O'Roak and State, 2008). ASD genes as a group are preferentially expressed in late mid-fetal prefrontal cortex and have concentrated expression in layer V/VI cortical projection neurons (Willsey et al., 2013). Although the developmental profile of GAT-1 in the mammalian brain is unclear, it is likely that GAT-1 plays an important role in early brain development by affecting GABA signaling. It is intriguing that our *in vitro* study in patient induced pluripotent stem cells (iPSC) detected a very low level but specific GABA uptake (Mermer et al., 2021). Although there were no gross abnormalities in the GAT-1 knockout mice (Jensen et al., 2003), the altered functionality of GAT-1 could affect brain circuitry, leading to autism. Collectively, studies from both rare monogenic mutations and common variants highlight the relevance of early fetal brain development in the pathophysiology of ASD.

Similar to autism, GE is also prominent childhood disorder and has a high co-morbidity with learning disability and cognitive impairment. GE includes a wide array of seizure phenotypes including childhood absence epilepsy (CAE), juvenile myoclonic epilepsy (JME), pure febrile seizures (FS), generalized epilepsy with febrile seizures plus (GEFS+) and generalized epilepsy with tonic–clonic seizures (GTCS). These GE syndromes vary greatly in severity from simple CAE that remit with age, to more severe phenotypes that involve intractable seizures and mental impairment like Dravet syndrome. It is well established that autism and epilepsy are often comorbid with each other. With genetic sequencing, mutations in various genes have been associated with epilepsy as well as autism. Up to date, there are at least ~80 established epilepsy genes and ~1,000 genes associated with epilepsy (Wang et al., 2017).

### Impaired GABAergic signaling gives rise to both autistic spectrum disorders and epilepsy

It is well established that compromised GABAergic signal pathway underlies both autism and epilepsy (Kang and Barnes, 2013). This is unsurprising considering the critical role of GABA signaling in early brain development (Maric et al., 2001; Cancedda et al., 2007). It is known that newly born cortical neurons undergo extensive migration before reaching their destination in the brain. GABA receptor activation influences the migration of immature cortical neurons (Owens and Kriegstein, 2002). GABA is synthesized from glutamic acid, the principal excitatory neurotransmitter, through a decarboxylation reaction catalyzed by glutamic acid decarboxylase. GABA activates two major types of receptors: one is GABA_A_ and the closely related GABA_C_ receptors, which function as chloride channels; the other type is the GABA_B_ metabotropic receptors.

GABA is a neurotrophic signaling compound, and it can directly impact neuronal differentiation, proliferation, and synaptogenesis during brain development. In embryonic and neonatal brains, GABA, via GABA_A_ receptors and GABA_B_ receptors, produces excitatory actions and acts as a trophic factor during nervous system development. It has been shown that GABA has multiple modes of actions at different developmental stages (Represa and Ben-Ari, 2005). GABA has a neurotrophic action in early brain developmental stages when networks are non-existent, and neurons consist of immature cells that have little communication. When GABAergic synapses begin to operate, the emerging network generates a coherent pattern of activity (Deykin and MacMahon, 1979). Mutations in GABA receptors or GAT-1 can disrupt GABA signaling, leading to lasting effects on neural circuitry and brain function. In adult brains with established neuronal networks, GABA_A_ receptors primarily mediate inhibitory neurotransmission, while GABA transporters facilitate GABA reuptake from the extracellular space. A malfunctioning receptor or transporter can impair neuronal inhibition through mechanisms specific to their respective functions. GABA interacts with both GABA_A_ and GABA_B_ receptors, and functional defects in either receptor can hinder GABA’s neurotrophic influence on neuronal development. Notably, mutations in GABA_B_ receptors have been linked to epilepsy and neurodevelopmental delays (Cediel et al., 2022). But this review paper will focus on mutations in GABA_A_ receptors because the mutational effect has been extensively researched in GABA_A_ receptors, and studies of mutant protein trafficking for GABA_B_ receptors are not available.

Mutations in various GABA_A_ receptor subunits are associated with autism (Table 1), frequently co-occur with epilepsy. Among the 19 members of GABA_A_ receptor super family, our work focuses on *GABRB3* that encodes the β3 subunit as it is more likely been associated with autism in addition to epilepsy in humans (Delahanty et al., 2011). GABA_A_ receptor subunits exhibit unique spatial and temporal distribution within CNS. Some subunits are globally expressed while others are only expressed in selected brain regions. For example, the α1 and γ2 subunits are widely expressed in the brain but the α5 subunit is only highly expressed in the hippocampus with a minimal level of expression in other brain regions (Laurie et al., 1992; Wisden et al., 1992). Additionally, for a given subunit, different splice isoforms may be differentially expressed. This is true for the β3 subunit as its transcription variant 2 is abundant in developing brain with minimal expression in the adult brain (Kirkness and Fraser, 1993). GABA_A_ receptors play a role in proliferation, migration, and differentiation of precursor cells that orchestrate the development of distinct regions of the embryonic brain (Barker et al., 1998). GABA as a neurotrophic signal, modulates neuronal arbor elaboration and differentiation. The neurotrophic role of GABA is also evidenced by the fact that GABA_A_ receptor antagonists reduced the dendritic outgrowth of cultured rat hippocampal neurons. Conversely, exposure to GABA increased the length and branching of the neurites and augments the density of synapses. The trophic action of GABA showed similar results in various studies and experimental models including cerebellar granule cells (Maric et al., 2001), cortical plate and subplate interneurons (Cancedda et al., 2007), spinal cord cells (Owens and Kriegstein, 2002) and raphe nuclei 5-hydroxytryptamine (serotonin)-producing neurons (Represa and Ben-Ari, 2005). The trophic effects of GABA have been reproduced by agents acting on GABA synthesis, receptor activation or blockade, intracellular Cl- homeostasis, or L-type Ca^2+^ channels. Furthermore, blockers of Ca^2+^/calmodulin kinase II (CaMKII) or mitogen-activated protein kinase reduce the trophic effects of GABA (Maric et al., 2001), suggesting an important role of Ca^2+^ influx and the activation of Ca^2+^ − dependent kinases in the neurotrophic effect mediated by GABA. In excitatory newborn neurons, an aberrant conversion of GABA-induced excitation/depolarization into inhibition/hyperpolarization leads to significant defects in synapse formation and dendritic development *in vivo* (Hartman et al., 2006; Borodinsky et al., 2003). Thus, GABA signaling is crucial for the establishment of synaptic contact and the regulation of neuronal activity (Hartman et al., 2006).

**Table 1:** Genetic variability in GABA_A_ receptor subunits associated with autism.

Gene	Variant count	Variant types	Evidence strength	Key notes
GABRB3	24 + SNVs + CNVs in 1–3% of ASD cases	22 rare exonic/regulatory variants ≥ 2 de novo coding (LoF/missense) CNVs (Dup15q)	Very High	CNVs among most common genetic events in ASD; missense and promoter variants directly linked
GABRA1	~8–10	De novo missense (e.g., R214C/H, T292S/I)	High	Strong co-occurrence of ASD with epilepsy and intellectual disability; electrophysiologically confirmed
GABRG2	~9–10	De novo missense (e.g., A106T, P282S/T, R323Q/W, P83S)	High	Strong link to epileptic encephalopathy with ASD traits; surface trafficking + channel dysfunction
GABRA4	~10 + SNPs	Intronic and upstream SNPs	Moderate	SNPs interact with GABRB1 in ASD families; KO mice exhibit social deficits
GABRB1	Several (unclear total)	Common SNPs	Moderate	Identified in GABRA4–GABRB1 interaction networks in ASD trios
GABRB2	≥5	Rare SNPs and missense	Moderate	ASD association found; also appears in schizophrenia; exact impact less defined
GABRA5	CNV only	Included in Dup15q syndrome	Indirect	ASD association via CNV duplication of 15q11–13, not isolated coding mutations
GABRG3	CNV only	Included in Dup15q	Indirect	Partnered with GABRB3/GABRA5 in CNV events
GABRA2/3/6	0	—	None	No ASD-linked sequence or structural variants reported
GABRD	0–2	Sparse case reports	Weak	Isolated epilepsy cases; no reliable autism associations
GABRE	0	None	None	Not implicated in ASD-related disorders

GABA plays critical roles in brain development via regulating neuronal differentiation, proliferation, and synaptogenesis. Conversely, impairment of GABAergic function in any of these epochs could potentially alter neural maturation, function, and circuitry, leading to the development of an excitable phenotype in the brain. For example, the neuronal morphological maturation in the somatosensory cortex was markedly impaired after blockade of GABA_A_ receptor function (Cancedda et al., 2007). GABRG2(R43Q, also numbered as R82Q by including the signal peptide mutation associated with FS and CAE) has been proposed to alter brain plasticity in the mutation knockin mice. This is evidenced by the observation that conditional suppression of the mutant allele in knockin mice from conception until postnatal day 20 decreases the susceptibility to pentylenetetrazole seizures in adulthood, when compared to mice with life-long expression of GABRG2(R43Q) allele. This suggests that disruption of GABA_A_ receptor activity during the sensitive brain development periods such as in fetal or neonatal brains may compromise the developmental processes, which could potentially result in the development of epilepsy (Ma et al., 2005). However, the detailed pathophysiological mechanisms of GABA_A_ receptor subunit mutation associated with human neuropsychiatric disorders on neuronal development, synaptogenesis and the complex network wiring, is still unclear.

### Mutations in *GABRB3* are associated with autism and epilepsy

There are many mutations in GABA_A_ receptors that are associated with epilepsy, but mutations in *GABRB3* than encodes β3 subunit of GABA_A_ receptor are more likely associated with autism (Table 1). Thus we focused on *GABRB3* in this study. *In vivo* deficiency of the GABA_A_ receptor β3 subunit is known to result in both epilepsy and a wide range of developmental abnormalities in the brain. To study this condition as a model for human epilepsy, *GABRB3* deletion mice were created. However, it has been frequently observed that these heterozygous gene deletion knock-out animals usually do not adequately mirror human loss of function epilepsy mutations and display no or a much milder phenotype than expected (DeLorey et al., 2008). For example, homozygous *GABRA1* gene deletion knockout mice only manifest tremors while heterozygous mice are behaviorally normal (Sur et al., 2001). Similarly, *GABRG2* heterozygous knockout mice display hyper-anxiety and are seizure-free. We have characterized the mechanisms underlying the phenotypical differences between the loss of function knockout and knock in mice and hypothesize at least two possible contributing factors. First, there is possible compensation by other functionally overlapping subunits for the loss of *GABRA1* and *GABRG2* subunits. Second, there are additional molecular risk factors involved in the pathogenesis of these mutations such as the disturbed signaling inside ER, which exacerbates disease phenotype. This notion has been validated in a comprehensive comparison between the *Gabrg2^+/−^* and Gabrg2*^+/Q390X^* mice at multiple levels from mRNA, protein assembly, EEGs, and neurobehavioral phenotypes (Warner et al., 2016). Comparing with the *Gabrg2^+/−^* mouse without the aggregation prone mutant γ2(Q390X) subunit protein only exhibiting infrequent absence seizures, the *Gabrg2^+/Q390X^* mouse recapitulates the major features of Dravet syndrome including increased mortality, spontaneous generalized tonic clonic seizures and impaired cognition (Kang et al., 2015; Warner et al., 2016; Warner et al., 2019).

Although G*ABRB3* knockout mouse has long been proposed to use as a model to study autism or similar neurodevelopmental disorders like Angelman syndrome, the role of *GABRB3* subunit in neurodevelopment and synaptogenesis is still unclear. In embryonic stem cells, β3 subunit is involved in cell proliferation. Activation of GABA_A_ receptors leads to hyperpolarization, increased cell volume and accumulation of stem cells in S phase, thereby causing a rapid decrease in cell proliferation (Andäng et al., 2008). In *GABRB3* KO mice, GABA mediated IPSCs are abolished in neurons from reticular thalamus, while the GABA mediated IPSCs from ventrobasal nuclear complex are unchanged. This data suggests that there is region-specific subunit distribution and argues for the brain region and subunit specific contribution of GABA_A_ receptors, which consequently affect specific neural circuit.

### GABA_A_ receptor β3 subunit signal peptide mutation GABRB3(P11S) associated with autism and epilepsy

As forementioned, many mutations in several GABA_A_ receptor subunits are associated with epilepsy. These GABA_A_ receptor subunits include but are not limited to *GABRA1, GABRA3, GABRA5, GABRA6, GABRB1-3, GABRG2* and *GABRD*. Among all the GABA_A_ receptor subunits, genes clustered in 15q11-q13 are associated with autism (Adak et al., 2023; Adak et al., 2021). The only autism associated mutation that has been characterized for function and trafficking is GABRB3(P11S). GABRB3(P11S) is a signal peptide variant in the β3 subunit variant 2. The mutation is associated with multiple pedigrees in Caucasian families exhibiting autism and childhood absence epilepsy. We have characterized the mutation *in vitro* and identified reduced GABA evoked current in the mutant receptors (Delahanty et al., 2011). The reduced GABA current is likely due to the reduced cell surface GABA_A_ receptor expression. More study is needed for further elucidating the disease mechanisms associated with this mutation.

### Mutations in GABA transporter 1 (GAT-1)-encoding *SLC6A1* are associated with autism and epilepsy

Mutations in the GAT-1 encoding *SLC6A1* gene are frequently associated with myoclonic atonic epilepsy and neurodevelopmental delay (Mermer et al., 2021; Wang et al., 2020; Mermer et al., 2022; Carvill et al., 2015). Based on previous studies (Johannesen et al., 2018; Goodspeed et al., 2020), more than 90% of *SLC6A1* patients exhibit epilepsy, and >80% exhibit developmental delay and cognitive impairment. About 20% of patients exhibit autistic traits. In brief, epilepsy, autism and neurodevelopmental delay were the most common clinical features (Table 2). We report 2–4 Hz spike wave discharges are frequently observed in patients associated with *SLC6A1* mutations (Wang et al., 2020; Mermer et al., 2022; Poliquin et al., 2021). Generalized epileptiform discharges were the most reported EEG abnormality (Goodspeed et al., 2020). Generalized background slowing and occipital intermittent rhythmic delta activity (OIRDA) is also reported (Poliquin et al., 2021). Recently, an exome-wide trio sequencing study identified *de novo* missense variants in *SLC6A1* to be associated with schizophrenia (Rees et al., 2020). In this small set of patients, there is no evidence of epilepsy, intellectual disability, or autism spectrum disorders that was reported. Besides various epilepsy syndromes, mutations in *SLC6A1* are also associated with autism.

**Table 2:** SLC6A1 variants associated with autism.

Protein variant	cDNA change	AA position	Variant type	Clinical features	ASD/autistic features	Source
p.Arg232His	c.695G > A	232	Missense	Developmental delay	Autism traits	Cai et al. (2019)
p.Gly234Ser	c. 700 G > A	234	Missense	Severe epilepsy (LGS)	Autistic features noted	
p.Arg251Gln	c.752G > A	251	Missense	Seizures	Autism spectrum symptoms	Goodspeed et al. (2020)
p.Ile268Serfs*36	c.801delC	268	Frameshift	Family with variable severity	ASD (proband nonverbal), ID	Clinical reports
p.Ala288Val	c.863C > T	288	Missense	Absence epilepsy, developmental regression	ASD diagnosis	Carvill et al. (2015)
p.Thr305Ile	c.914C > T	305	Missense	Myoclonic-atonic epilepsy	ASD features	Wang et al. (2020)
p.Pro361Thr	c.1081C > A	361	Missense	Generalized epilepsy	Clinically diagnosed ASD	Wang et al. (2020)
p.Arg347Gln	c.1040G > A	347	Missense	Cognitive impairment	Autism	Epi4K Consortium, 2013
p.Val342Met	c.1024G > A	342	Missense	Developmental delay, epilepsy (siblings)	Autistic features in sibs	SLC6A1 Connect
p.Gly297Arg	c.889G > A	297	Missense	Myoclonic-atonic seizures	Autistic traits noted	Carvill et al. (2015)
p.Gly550Arg	c.1648G > A	550	Missense	Epilepsy, intellectual disability	Autism	Johannesen et al. (2018)

We previously reported the GAT-1(P361T) mutation as being associated with epilepsy and autism in a Chinese cohort (Wang et al., 2020). We also reported a recurring mutation A288V with epilepsy and autism that has been identified in other cohorts (Carvill et al., 2015). Others have reported GAT-1(A288V) in autism as well as in absence epilepsy, myoclonic atonic epilepsy, and neurodevelopmental delay (Carvill et al., 2015; Johannesen et al., 2018). We have studied the molecular pathophysiology of both GAT-1(A288V) and GAT-1(P361T) mutations. In contrast to the extensively studied GAT-1(S295L) that causes almost a complete loss of function, both GAT-1(A288V) and GAT-1(P361T) mutations caused a partial loss of function with ~20 to 35% remaining GABA uptake activity. In cell models, both mutation cause the ER retention of the mutant protein but to a lesser degree compared with the mutant GAT-1(S295L) transporter 1 protein associated with childhood absence epilepsy and neurodevelopmental delay. The impact of the mutation may be more complicated *in vivo* considering the interaction of the wildtype and the mutant alleles in both GAT-1(A288V) and GAT-1(P361T) mutations while the mutant GAT(S295L) may be simply degraded (Wang et al., 2020; Mermer et al., 2022).

### Common ER retention and abnormal subcellular compartment distribution for GABA_A_ receptors and GAT-1

ER is a continuous membrane system that forms a series of flattened sacs within the cytoplasm of eukaryotic cells and serves multiple functions. ER is essential for protein synthesis, folding, modification, maturation, and transport to desired destination. In animal cells, the ER usually constitutes more than half of the membranous content of the cell. ER can be classified as rough ER and smooth ER based on certain physical and functional characteristics. Rough ER is named for its rough appearance, which is due to the ribosomes attached to its outer (cytoplasmic) surface. Rough ER lies immediately adjacent to the cell nucleus, and its membrane is continuous with the outer membrane of the nuclear envelope. The ribosomes on rough ER specialize in the synthesis of proteins that possess a signal sequence that directs them specifically to the ER for processing. Smooth ER is more tubular than rough ER and forms an interconnecting network sub-compartment of ER. Smooth ER is devoted almost exclusively to the manufacture of lipids and in some cases to the metabolism of them and associated products. Smooth ER is less related to this study so it will not be discussed. Proteins synthesized by the rough ER have specific final destinations such as cell membrane. Membrane proteins like GABA_A_ receptor subunits need to be properly folded and modified by glycans, assembled inside ER lumen before secreted to the Golgi apparatus and then directed to lysosomes or to the cell membrane.

Molecular chaperones are proteins that assist the correct non-covalent assembly of other proteins to adopt proper conformation, but which are not components of these assembled structures such as GABA_A_ receptor when they exert their biological functions. Numerous ER-resident chaperones and enzymes aid in structural and conformational maturation necessary for proper protein folding, including signal-peptide cleavage, N-linked glycosylation, disulfide bond formation, and glycophosphatidylinositol (GPI)-anchor addition (Ellgaard and Helenius, 2003). These processes are compromised in GABA_A_ receptor subunit mutations in various ways but often manifest as increased ER retention and enhanced degradation of the mutant protein and, in some cases, the partnered wildtype GABA_A_ receptor subunit *in vitro* (Kang et al., 2009a,b). Our previous study indicates that the mutant GABA_A_ receptor γ2(Q390X) subunit increased binding with the chaperones such as Calnexin and Binding immunoglobulin protein (Bip) (Shen et al., 2020), suggesting the mutant GABA_A_ receptors may trap the molecular chaperones and reduce their function.

We have extensively studied the mutant protein subcellular localization for both GABA_A_ receptor and GAT-1 mutations. The ER uses an elaborate surveillance system called the ER quality control system. This quality control system facilitates folding and modification of secretory and membrane proteins and eliminates terminally misfolded polypeptides through ER-associated degradation (ERAD) (Kang and Macdonald, 2016) or autophagic degradation (Kim et al., 2022). Cells ensure efficient and accurate production of secretory and membrane proteins and constantly maintain proper physiological homeostasis in the ER including redox state and calcium balance via the elaborate quality and quantity control systems.

For GABA_A_ receptor mutations, we identified mutant protein ER retention with or without detectable change of ER stress hallmark, the growth arrest- and DNA damage-inducible gene 153 (GADD153). The ER retention and enhanced mutant protein degradation has been identified across GABA_A_ receptor subunits including *GABRA1* (Gallagher et al., 2005)*, GABRB1, GABRB2* (Ishii et al., 2017), *GABRB3* (Shi et al., 2019), *GABRA3, GABRA5, GABRA6* and *GABRG2* (Kang et al., 2009a). Among all the surveyed mutations, the GABRG2(Q390X) associated with a severe type of epilepsy termed Dravet syndrome, has been most extensively characterized. We identified protein ER retention (Kang et al., 2009a), ER stress (Shen et al., 2020), chronic accumulation of the mutant protein *in vivo*, disturbed chaperone activity and increased neuroinflammation in the knockin mice (Kang et al., 2015).

For mutant GAT-1 proteins, we have studied a large array of mutations in patients (Mermer et al., 2021; Mermer et al., 2022). These mutations are associated with a wide spectrum of disease phenotypes including myoclonic atonic epilepsy, absence epilepsy, autism, neurodevelopmental delay, and intellectual disability. Interestingly, all the mutations displayed either a partial or a complete loss of GABA uptake function (Mermer et al., 2021; Wang et al., 2020; Mermer et al., 2022; Poliquin et al., 2021; Nwosu et al., 2022). Most of the mutations displayed ER retention, a subcellular localization profile similar to cells expressing the wildtype but treated with ER stress inducer tunicamycin (Wang et al., 2020; Poliquin et al., 2021). We conducted a critical analysis of the subcellular localization of mutant GAT-1 in neurons and astrocytes that were derived from human induced pluripotent stem cells and mutation knockin mice (Mermer et al., 2021; Mermer et al., 2022). For example, the GAT-1(S295L) formed large ER-retained lumps in both mouse astrocytes and human astrocytes when transfected. This was not observed with GAT-1 in the wildtype cell models. The similar ER retention of the mutant GAT-1 is also observed in the human neurons derived from patient induced pluripotent stem cells(iPSCs). This thus suggests ER retention is common across *GABR* and *SLC6A1* mutations and across cell types for a give mutation. It is likely that the mutant GAT-1 protein is subject to the same principle for ER associated degradation and retention. A recent study with Drosophilia melanogaster on GAT-1 mutations as well as previous studies on other *SLC* transporters such as SLC6A8 associated with creatine deficiency also supports this notion of mutant protein ER retention for at least a subset of mutations (Kasture et al., 2022; El-Kasaby et al., 2019; Angenoorth et al., 2022).

### Reduced cell surface expression in both mutant GABA_A_ receptors and mutant GABA transporter 1 associated with epilepsy and autism

We have studied various mutations in GABA_A_ receptor subunits and GAT-1 encoding SLC6A1 and have since identified common molecular pathophysiology shared in both GABA_A_ receptor and GABA transporter 1. For GABA_A_ receptor mutations, we have studied mutations in *GABRA1, GABRG2, GABRB1, GABRB2, GABRB3, GABRA5* and *GABRD* subunits (Kang and Macdonald, 2016). We identified impaired membrane trafficking, ER retention, and altered channel gating kinetics as common patho-mechanisms. For mutations in GAT-1, we identified similar mechanisms as in GABA_A_ receptors. Importantly, we identified similar ER retention of the mutant protein in astrocytes (Mermer et al., 2021; Mermer et al., 2022). This is critical as the GAT-1 protein is expressed in both neurons and astrocytes. This also suggests pharmacological compounds can similarly alleviate the pathophysiology in both cell types.

### 4-Phenylbutyrate (PBA) rescues disease phenotypes in both *SLC6A1* and GABA_A_ receptor mutation mediated disorders

**Figure 2:**
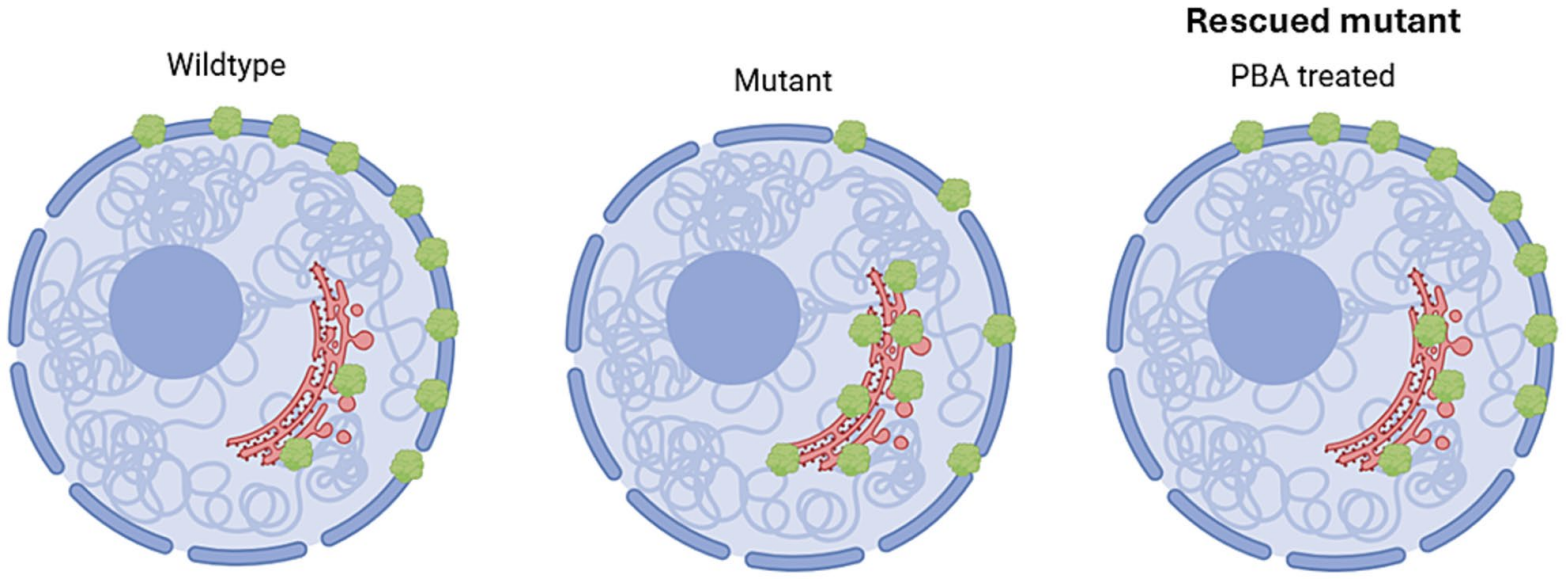
Rescuing mechanisms targeting endoplasmic reticulum protein retention for a subset of autism. Based on our studies on the mutant GABA_A_ receptor and GABA transporter 1, we propose that compared with the wildtype (Wildtype), the mutant (Mutant) protein associated with autism is more likely retained in the endoplasmic reticulum (ER). Chemical chaperones or pharmacochaperone such 4-phenylbutyrate (PBA) can enhance protein trafficking, enhance protein refolding and protein clearance, thus rescuing the mutant condition.

We have extensively tested PBA in GABA transporter 1 and GABA_A_ receptor mutations. We identified that PBA could restore GABA uptake for *SLC6A1* mutations in cell models including astrocytes and neurons differentiated from human patient iPSCs (Nwosu et al., 2022). More importantly, PBA alone mitigated seizures in the patient mutation bearing *Slc6a1^+/S295L^* knock-in mice. PBA is a Food and Drug Administration-approved drug for pediatric use, so its safety is time-tested. In addition, it is orally bioavailable. In a previously published study, we examined the impact of PBA across a library of variants in cell models, PBA restored the GABA uptake in most mutant homozygous conditions but not in those with premature stop codon generating mutations. However, in the heterozygous condition, it unanimously increased GABA uptake for both missense and nonsense mutations, suggesting its potential broad application. We have demonstrated the existence of the mutant GAT-1 suppressed the expression of wildtype *γ*-amino butyric acid transporter 1, suggesting the mutant protein causes aberrant protein oligomerization. In mice, we identified that PBA alone mitigated 5–7 Hz spike wave discharges in the *Slc6a1^+/S295L^* mice by 76% (Nwosu et al., 2022). More importantly, we demonstrated that PBA reduced seizures in both animal models of epilepsy and human patients (DeLeeuw et al., 2025). Although the action of mechanisms is not totally clear, the finding on the PBA effect in *SLC6A1* mutations is encouraging and will shed light on further treatment development. Importantly, we also demonstrated that PBA could mitigate seizures in the mouse model of Dravet syndrome associated with *GABRG2* mutation (Shen et al., 2024). Based on the studies in both GABA_A_ receptors (Shen et al., 2024) and *SLC6A1* mutations (Nwosu et al., 2022), PBA can increase the number of functional receptors or transporter 1on the cell surface and reduce the number of the nonfunctional receptors or transporter 1 inside the ER (Figure 2).

### Common therapeutic treatment options for autism and epilepsy are associated with monogenic mutations

**Figure 3:**
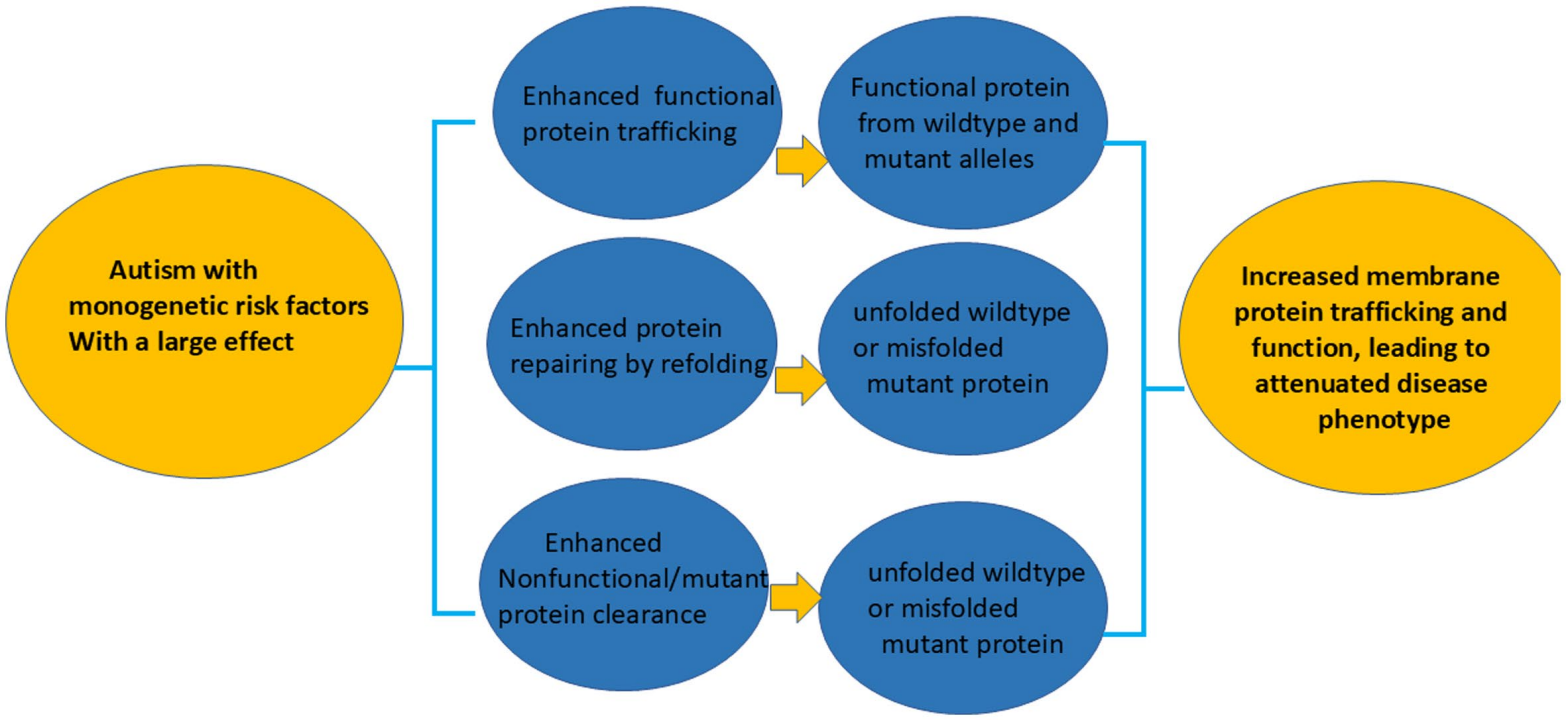
The rescuing mechanisms for the subset of autism with monogenic mutations via chemical chaperones or pharmacochaperones. Based on our studies of mutant GABA_A_ receptor and GABA transporter 1 (GAT-1), we propose three potential mechanisms to rescue the dysfunctional proteins associated with a subset of autism: (a) enhancing protein trafficking; (b) promoting protein refolding or repair; and (c) facilitating the clearance of nonfunctional or misfolded proteins. These interventions aim to increase the expression of functional proteins at the cell surface or synapse, thereby restoring the activity of disease-associated GABA_A_ receptors or GAT-1. An enhanced receptor or transporter function is expected to alleviate the disease phenotypes in autism caused by *de novo* monogenic mutations with large effects.

We propose that for the subset of autism and epilepsy caused by monogenic mutations, treatments like PBA, currently used for urea cycle disorders, or pharmacological chaperones developed for other conditions, could be repurposed as effective therapies (Figure 3). Based on our extensive studies on GABA_A_ receptor and GAT-1 mutation, there are common disturbed ER pathologies, which can be ameliorated by compounds such as PBA. This approach is disease modifying as the chaperones will assist in protein folding and increase the function of the diseased protein. This thus modifies the disease pathophysiology at the root level. At the behavioral level, autism and epilepsy involve different neuronal circuitries. For example, cortical-thalamic cortical circuitry has been established to play a significant role in absence epilepsy. Meanwhile, the repetitive behaviors in autism may involve a selective synaptic impairment in the nucleus accumbens/ventral striatum circuitry (Rothwell et al., 2014). However, at a root level, at least a subset of mutations in both disorders may cause ER retention and impaired membrane protein trafficking of GABAergic proteins. Thus, with the subset of autism that possesses molecular pathophysiology such as protein ER retention, reduced membrane trafficking may be a target for treatment. Chemical chaperones such as PBA or any pharmacochaperones that were developed for epilepsy that have demonstrated the ability to rescue trafficking deficient mutant protein such as caused by *SLC6A1* mutations (Nwosu et al., 2022) may be an effective treatment option for autism.

## Conclusion

Both autism and epilepsy can result from *de novo* monogenic mutations in various genes, including those encoding GABA_A_ receptors and the GAT-1. Within the ER, mutant proteins, regardless of the specific disorder or phenotype they cause, are likely to disrupt common ER networks and downstream cellular processes. We propose that at least a subset of autism and epilepsy share underlying molecular pathophysiology, particularly ER-related dysfunction caused by these mutant proteins. Consequently, therapeutic approaches developed for epilepsy may have potential for adaptation in treating autism.
